# Immune-Regulatory Mechanisms in Systemic Autoimmune and Rheumatic Diseases

**DOI:** 10.1155/2012/941346

**Published:** 2011-10-27

**Authors:** Yuya Takakubo, Yrjö T. Konttinen

**Affiliations:** ^1^Department of Medicine, Biomedicum Helsinki, University of Helsinki, P.O. Box 700 (Haartmaninkatu 8), 00029 HUS, Finland; ^2^Department of Orthopedic Surgery, Yamagata University School of Medicine, Yamagata 990-9585, Japan; ^3^Department of Medicine/Invärtes Medicin, Helsinki University Central Hospital, 00029 Helsinki, Finland; ^4^ORTON Orthopedic Hospital of the ORTON Foundation, 00281 Helsinki, Finland; ^5^COXA Hospital for Joint Replacement, 33101 Tampere, Finland

## Abstract

Systemic autoimmune and rheumatic diseases (SAIRDs) are thought to develop due to the failure of autoimmune regulation and tolerance. Current therapies, such as biologics, have improved the clinical results of SAIRDs; however, they are not curative treatments. Recently, new discoveries have been made in immune tolerance and inflammation, such as tolerogenic dendritic cells, regulatory T and B cells, Th 17 cells, inflammatory and tolerogenic cytokines, and intracellular signaling pathways. They lay the foundation for the next generation of the therapies beyond the currently used biologic therapies. New drugs should target the core processes involved in disease mechanisms with the aim to attain complete cure combined with safety and low costs compared to the biologic agents. Re-establishment of autoimmune regulation and tolerance in SAIRDs by the end of the current decade should be the final and realistic target.

## 1. Introduction

Recently, several important new discoveries have been made on both immunogenicity and autoimmune regulation and tolerance. Immune tolerance is necessary for the homeostatic and balanced host defense. It is important to recognize the role of the self-antigens which should be protected (non-dangerous) or self- and nonself-antigens which should be eliminated (dangerous) for the induction and maintenance of autoimmune regulation and tolerance. Immune system is constantly in contact with numerous self-antigens including autologous necrotic or apoptotic tissues, and it uses multiple strategies to prevent autoimmunity [1]. The antigen-specific or nonspecific immune tolerance can be generated primarily in thymus or secondarily in peripheral lymphatic and extralymphatic tissues. Various elimination mechanisms of autoreactive T cells and B cells such as presentation of autoantigen by antigen-presenting cells (APCs) and regulation by regulatory T cells (Tregs) play a role, not only for the central tolerance in the thymus, but also for the peripheral tolerance in extrathymic tissues, which exert an ongoing control to avoid systemic autoimmune and rheumatic diseases (SAIRDs). Within the thymus, T cells go through positive and negative selection processes in anatomically different locations to shape the entire peripheral T-cell repertoire when the central tolerance is established. Peripheral tolerance is then produced by engagement of dendritic cells (DCs) via several mechanisms, including generation and expansion of Treg cells and regulatory cytokines [2–5]. 

The onset and progression of SAIRDs depends on multiple factors, and many types of cells are involved in the multiple pathways of the immune reaction [5–7]. In all SAIRDs, the ultimate goal should be the re-establishment of self-tolerance [8]. In this paper, we describe new insight and topics related to immune regulation and tolerance in SAIRDs and their potential in the management of these diseases.

## 2. The Pathogenic Role of Dendritic Cells, Regulatory T and B Cells, and Regulatory Cytokines in SAIRDs

### 2.1. Dendritic Cells (DCs)

The functional abilities of the DCs vary depending on their subset and state of maturation. The two major categories of DCs are the conventional DCs (cDCs) and the plasmacytoid DCs (pDCs). They can be further functionally classified to mature and immature DCs. DCs for a heterogeneous group of cells, which play an important role in immunogenicity but also in the maintenance of immunotolerance, including their effects on the induction of antigen-specific T cell responses resulting in anergy, apoptotic deletion, or formation of Tregs [9]. Tolerogenic DCs populations have been generated as experimental therapeutic tools, which have been used with some success in murine disease models [10]. However, direct evidence implicating a particular DC subset in the breach of self-tolerance leading to SAIRDs is lacking although some novel murine experimental arthritis models allow delineation of early, preclinical events leading to the loss of self-tolerance [11, 12]. 

### 2.2. Conventional Dendritic Cells (cDCs)

CDCs are detected in blood, skin, secondary lymph nodes, spleen, and inflammatory synovitis [1, 4, 7, 13]. They are subdivided into two categories, migratory DCs and resident DCs, for example, Langerhans cells in the skin and mucosal membranes. In addition, there are other types of cDC that are derived from monocytes during inflammation [14–16]. 

In immune reactions mature cDCs play a more important role as an APC to activate naïve T cells than pDCs [12, 17]. In addition, these cells play a central role in the tumor necrosis factor (TNF) *α*-dependent experimental autoimmune arthritis initiated by an irrelevant nonarticular antigen and no other APC is by itself sufficient for breach self-tolerance mediated by endogenous pathways [8]. External (pathogen-associated molecular patterns) and internal (alarmins) danger signals or a combination of both leads to the maturation of cDCs in various inflammatory and infection diseases through membrane receptors, such as Toll-like receptors (TLR) 1-6, TLR8, and intracytoplasmic inflammasome [8, 12, 17].

On the other hand, engagement of immature cDCs with naïve T cells is thought to result in immunological tolerance. Immature cDCs are characterized by low surface expression of major histocompatibility complex- (MHC-) II and costimulatory molecules [9, 17]. Immature cDCs can drive naïve T cells to assume Tregs phenotype and/or promote the function of already existing Tregs as has been shown in experiments in which antigen was administered to mice without a concomitant maturation signal. Under these conditions, antigen accumulated on cDCs in secondary lymphoid organs and triggered the differentiation and/or proliferation of Tregs, resulting in antigen-specific tolerance that could prevent or reverse autoimmune processes [18–20].

### 2.3. Plasmacytoid Dendritic Cells (pDCs)

PDCs can function to limit self-reactivity and consequent pathology [11]. These cells are also known as interferon- (IFN-) *αβ* (type I IFN-) producing cells [11, 21]. Several systemic autoimmune diseases cause a prominent IFN signature (interferon-regulated genes) in the affected target tissue which pDCs produce, such as rheumatoid arthritis (RA), systemic lupus erythematosus (SLE), psoriatic arthritis (PsA), systemic sclerosis (SSc), and Sjögren's syndrome (SjS). PDCs are activated to produce type I IFN by recognition and internalization of immune complexes consisting of autoantibodies and self-nucleic-acids, which after endocytosis are recognized through TLR7 and TLR9. On the other hand, pDCs are reported to be key players in the establishment of oral and transplant tolerance. Human pDCs activated by the TLR9 DNA ligand CpG-ODN can induce Tregs [22, 23]. PDCs has the potential to express indoleamine 2,3-dioxygenase (IDO), an enzyme that via its products inhibits effector T cell proliferation in SAIRDs [13, 24]. In the presence of extracellular IDO, T cell proliferation is compromised, and adaptive differentiation Tregs is enhanced although the precise molecular basis for this effect is still unclear [20, 25, 26].

### 2.4. Regulatory T Cells (Tregs)

Adaptive effector T cell responses to self and nonself-antigens can be efficiently controlled by regulatory T cells belonging to the CD4+CD25+ Treg subset. Tregs are phenotypically heterogeneous and include both CD4+ and CD8+ T cells, most of which express forkedhead box p3 (Foxp3) [20, 27]. Tregs can express anti-inflammatory molecules, such as interleukin (IL)-10, transforming growth factor (TGF)-*β* and IL-35, and/or inhibitory receptors, such as cytotoxic T-lymphocyte antigen 4 (CTLA4), lymphocyte-activation gene 3 (LAG-3), glucocorticoid-induced tumor necrosis factor receptor (GITR), CD39, and CD73 [19, 28].

DCs constantly present innocuous self- and nonself-antigens in a fashion that promotes tolerance, at least in part, through the control of Tregs [29]. Failure of Tregs function has been implicated in the development of many autoimmune processes and, *vice versa*, cellular therapies by adoptive transfer of Tregs have shown efficacy in these disorders [20, 30]. In addition, DCs and Tregs regulate homeostasis of each other [18–20].

Natural Tregs (nTregs) in thymic tissue develop and maintain central tolerance during the fetal period. Conventional naïve T cells can develop to so-called adaptive Tregs (aTregs) in extrathymic tissues such as secondary lymphoid organs. This is important, because the autoimmune T cell have not been completely deleted by the central tolerance mechanisms. Therefore, aTregs play a complementary role in the maintenance of the peripheral tolerance by regulating peripheral autoimmune T cells. The role of these inflammation-induced aTregs is not fully understood, but they seem to limit immunohistopathologic changes by suppressing autoaggressive responses and/or by promoting restitution of tissue homeostasis (via TGF-*β*) or T and B cell memory (via IL-10). Antigen-specific Tregs can spread their tolerance-promoting capacity to local DCs and naïve T cells through a mechanism called “infectious tolerance”, which means that tolerant T cells, which are incompletely stimulated by APCs, induce new naïve T cells toward a similar state of tolerance [20, 27, 31].

### 2.5. Regulatory-B Cells (Bregs)

The efficacy of B cell suppression with anti-CD20 therapy such as Rituximab in the treatment of RA indicates an important role for B cells in the pathogenesis of RA. In RA, transient B cell depletion with Rituximab can ameliorate disease for a prolonged period but typically not indefinitely. Autoreactive B cells have been found in bone marrow and peripheral tissues [32]. Anti-TNF agents decreased the peripheral blood memory and germinal center B cells in RA patients restoring the early B cell tolerance in RA [33]. In addition, B cells are the main producers of LT*α* and an important producer of TNF*α* in RA synovitis. B cells can also secrete multiple other cytokines, including IL-1, IL-4, IL-6, IL-7, IL-8, and IL-12, granulocyte colony-stimulating factor (G-CSF), and granulocyte-macrophage colony-stimulating factor (GM-CSF) [34, 35]. 

On the other hand, B cells are able to suppress autoimmunity in various animal models through production of IL-10 and/or TGF-*β* and by cytokine-independent mechanisms. Murine models have also revealed a suppressive role for B lymphocytes. Following stimulation of CD40, B cells inhibit the induction of arthritis through secretion of IL-10 [36]. In Palmerston North SLE mouse model, regulatory B cells (Bregs) produce high levels of IL-10 that inhibit production of IL-12, which leads to diminished inflammatory response to bacterial DNA [37]. A role for Bregs has been reported in a mouse model of inflammatory bowel disease and experimental autoimmune encephalomyelitis [38, 39]. Depending on the model studies, antigen-specific autoreactive T and B cells, DCs, macrophages and/or Tregs are regulated by the Bregs populations [40]. These observations contribute to the new and important concept of Bregs [32, 41].

## 3. Advances in Molecular Genetics and Molecular Diagnostics

### 3.1. Genetics in Systemic Autoimmune and Rheumatic Diseases (SAIRDs)

Advances in the field of human genetics have led to a rapid increase in the number of new disease risk alleles and loci identified in SAIRDs patients. In particular, genes involved in the nuclear factor (NF)-*κ*B pathway and T cell-DC interactions seem to be involved in SAIRDs [42–44]. 

New candidate genes have been reported in RA, and they include single-nucleotide polymorphisms (SNPs) of IL-6, signal transducer and activator of transcription (STAT) 4, IL-2RA, CC chemokine ligand (CCL) 21, CD40, CD244, TNF alpha-induced protein (TNFAIP) 3, sprouty-related protein with enabled/vasodilator stimulated phosphoprotein homology 1 domain (SPRED) 2, recombination signal binding protein for immunoglobulin kappa J region (RBPJ), CC chemokine receptor (CCR) 6, interferon-regulatory factor (IRF) 5, PX domain containing serine/threonine kinase (PXK), cyclin-dependent kinase (CDK) 6, and vitamin D (Vit.D) Fokl found in genome-wide association studies (GWAS) [42, 43, 45, 46]. The heritability of RA has previously been estimated to be about 60%. Recently, it was reported that the heritabilities of anticitrullinated protein antibody- (ACPA-) positive and ACPA-negative RA are rather similar, 68% and 66%, respectively [47–50]. In particular, human leukocyte antigen- (HLA-) DRB1 and shared epitope (SE) alleles of it were reported the strong association with ACPA-positive RA. Apart from perhaps gender, the main known genetic factor predisposing to RA is HLA and its contribution to the genetic variation has previously been estimated to be 37% [51].

In SLE, the IRF5 haplotype has been reported to relate to an increased production of type I IFN with a concomitantly increased risk for SLE [52], and a variant of STAT4 increases the sensitivity to type I IFN in patients with SLE [53]. A variant of TNFAIP 3, which encodes an ubiquitin-editing enzyme inhibiting NF-*κ*B-dependent signaling and prevents inflammation, and polymorphic haplotypes of the human T-lymphotropic virus*-*1-related endogenous sequence (HRES) 1 long terminal repeat (LTR) have been associated with SLE [54, 55]. New candidate changes include SNPs in CD40 region, B-lymphoid tyrosine kinase (BLK) gene, vascular endothelial growth factor (VEGF) receptor 2 gene, and Vit.D Bsml gene [44, 45].

Polymorphisms in IRF5 are not only associated with RA and SLE, but also with SSc, inflammatory bowel diseases and multiple sclerosis. An association with STAT4 was first identified in RA and SLE, but later also found in SSc, inflammatory bowel diseases, type 1 diabetes, psoriasis, and primary antiphospholipid antibody syndrome [44].

GWASs have disclosed over twenty candidate genes in PsA, including IL-4, IL-12B, IL-13, IL-23A, IL-23R, TNFAIP3, TNFAIP3 interacting protein (TNIP) 1, TNF receptor-associated factor (TRAF) 3 interacting protein (IP) 2, nitric oxide synthase (NOS) 2, v-rel reticuloendotheliosis viral oncogene homolog (REL), endoplasmic reticulum aminopeptidase (ERAP) 1, F-box and leucine-rich repeat protein (FBXL) 19, NF-*κ* light polypeptide gene enhancer in B cells inhibitor, alpha (NFKBIA), interferon induced with helicase C domain (IFIH) 1, IL-28RA, and tyrosine kinase (TYK) 2 [56, 57].

In SSc, the newly reported candidate genes and risk loci include CD247, MHC, IRF5, and STAT4 gene regions, psoriasis susceptibility 1 candidate (PSORS1C) 1, TNIP1, and putative one close to *ras* homolog gene family, member B (RHOB) gene [58]. 

In SjS, the newly reported candidate genes and risk loci include early B-cell factor 1 (EBF1) gene, family with sequence similarity 167 member A (FAM167A)-BLK locus, TNF superfamily4 gene, HLA-DRB1∗0301, -DQA1∗0501, and -DQB1∗0201, with SNPs in IL-10 promoter, Fas/FasL, TGF-*β*1, and TNF*α* genes, IRF5 and STAT4 [44].

In addition to the genetic factors also environmental risk factors and their interactions with the genetic factors contribute to SAIRDs, in particular to RA [42]. One important environmental risk factor for RA is smoking, which increases the odds ratio to about 1.8 [59]. SE alleles, lymphoid-specific tyrosine phosphatase (LYP)/protein tyrosine phosphatase, nonreceptor type (PTPN) 22, and cigarette smoking are risk factors for the development of ACPA positive RA patients [47, 60]. Further, it has been reported that also the noninherited maternal HLA antigens (NIMA), originally discovered in transplantation immunology [61], containing the RA-protective DERAA sequence, protect DERAA-negative children against RA [49, 62]. 

It has been recently reported that the gene polymorphism may not only be associated with the pathogenesis of SAIRDs, but also with the outcome of the treatment and the progression of the disease. GWAS and new generation sequencing disclosed that the response of RA patients to anti-TNF therapy was associated with ATP-binding cassette, subfamily A (ABCA) 1, solute carrier family 44 (SLC44A) 1 gene and granzime B SNP were associated with joint destruction, and protein tyrosine phosphatase, receptor type, C (PTPRC) locus (rs10919563) and TRAF 6 [63–67].

### 3.2. MicroRNA

MicroRNAs have attracted attention as potential new biomarkers useful for the diagnosis of early disease and for the prediction of drug response in SAIRDs. MicroRNAs form an abundant class of endogenous, short, noncoding single stranded RNA molecules (19–23 nucleotides) that function as posttranslational regulators of the expression of genes, such as inflammatory cytokines. MicroRNAs were first reported in cancer [68]. Recently, many microRNAs, such as microRNA-16, -146a, -155, and -223, have been detected in SAIRDs samples, and it is thought that their expression regulates disease onset/activity and drug responses [68, 69].

## 4. New Insights into Defective Regulatory Function in SAIRDs

### 4.1. Toll-Like Receptor (TLR) and Interferon (IFN) Signature

In the last decade, a relationship has been described between TLR and IFN signature. IFN signature is refers to a pattern of genes regulated by IFN in inflamed target tissues. IFN signature is typical for many SAIRDs, such as RA, SLE, and SSc. DNA- or RNA-containing immune complexes, formed as a result of autoantibodies binding antigenic material derived from apoptotic or necrotic cells, can act as endogenous type I IFN inducers [70–72]. Prolonged exposure of the immune system to type I IFN increases the risk for the loss of self-tolerance and for subsequent autoimmune reactions. Indeed, high-serum type I IFN serum levels have been associated with the development of SAIRDs like SLE [73–75]. 

PDCs are activated to produce type I IFN when they recognize immune complexes consisting of autoantibodies and self-nucleic acids. Following Fc*γ*RIIa- (CD32-) mediated internalization, they trigger endosomal TLR7 and TLR9 [76, 77]. PDCs are low in numbers in the blood of SLE patients, but large amounts of type I IFN are in lupus produced to serum by the activated pDCs located in the lymph nodes, skin, and other target organs [74, 78]. Proper clearance of apoptotic cell rests is normally thought to prevent harmful exposure of the immune cells to self-antigens and alarmins and to prevent their autoaggressive activation [16, 79]. Type I IFN plays powerful roles in shaping immune responses in SAIRDs by activating lymphocytes, macrophages, DCs, and natural killer (NK) cells with the expression of IFN signature [80–82]. If apoptotic cells are not adequately cleared, condensed, and fragmented cell rests enter late stage apoptosis or undergo secondary necrosis, which causes release of nucleosomes, small nuclear ribonucleoproteins (snRNPs), and DNA, but also high-mobility group box 1- (HMGB1-) nucleosome complexes and other signals able to trigger inflammation through TLRs [83–85]. Only extracellular free HMGB1 or nucleosomes alone were not able to activate DCs or induce cytokine production. HMGB1 was thought to inhibit and diminish the phagocytosis of apoptotic neutrophils by macrophage through binding to phosphatidylserine on the neutrophil membrane and the remains of them might activate to produce proinflammatory mediators through TLR2 or TLR4 [86]. From studies of lupus-prone mice, it appears that there are multiple defects in the process that regulates autoreactive T and B cells, allowing the maturation of autoreactive B cells. In SLE patients, several abnormalities interfere with the clearance of apoptotic cell rests, microparticles and chromatin, such as complement defects [87] and reduced levels and activity of DNase I in serum [88]. In lupus-prone mice model, a deficiency in a negative regulator (Tir8/Sigirr) of TLR signaling accelerates disease progression [89]. Some TLR7-deleted lupus-prone mice strains have decreased lymphocyte and pDC activation, decreased serum IgG, and ameliorated autoimmune disease [90]. TLR7-dependent production of autoantibodies is decreased in such mouse strains. In contrast, although CpG-containing DNA, which is a ligand of TLR9, is induced lupus-like disease, lack of TLR9 in the MRL/lpr mouse background exacerbated autoantibody production and disease activity, suggesting that TLR9 protects against lupus in this model [90, 91]. TLR7 and TLR9 appear to have different roles in the development of murine lupus. Taken together, these studies suggest TLR9 may either protect or exacerbate autoimmune conditions depending on the genetic background of the lupus-prone mice. Jin et al. demonstrated that peripheral circulating pDCs in patients with SLE were functionally abnormal and that they lacked TLR9 [23]. The role of TLR9 in SLE development is still unclear.

### 4.2. Th17 and Regulatory T Cells (Tregs)

Th 17 cells produce IL-17 (or actually IL-17A), IL-21 and IL-23 and have been reported to play a crucial role in inflammation in SAIRDs [92, 93]. A combination of IL-6 and TGF-*β* is required for the development of Th 17 cells [92]. Apoptotic bodies and nucleosomes induce maturation of mouse cDCs to produce high amounts of IL-6 [79]. On the other hand, the development and maturation of Tregs is inhibited by IL-6 [15, 94, 95]. IL-6 produced from LPS-treated DC activated effector T cells and blocked the suppressive effects of Tregs [94]. In fact, SLE patients have significantly lower numbers of CD4+CD25+ Tregs than healthy controls [23, 96]. Anti-TNF therapy increased Foxp3 mRNA and protein expression by Tregs and restored their suppressive function [97].

Recently, it was shown that retinoic acid, which binds the nuclear retinoic acid receptor *α* (RAR*α*), increases the expression of Foxp3 and Smad3 in T cells and inhibits the generation of Th 17 cells [98]. Retinoic acid may be a candidate drug for RA therapy, because alltrans-retinoic acid (ATRA) improves clinical symptom in collagen-induced arthritis mice [99].

### 4.3. Interleukine (IL)-27 and IL-35

IL-27 and IL-35 are the dominant anti-inflammatory cytokines. IL-27, a member of the IL-12 family, is a heterodimeric cytokine consisting of Epstein-Barr virus-induced gene 3 protein (EBI3) and a unique IL-27-p28 [100]. In collagen-induced mice, IL-27 reduced disease development and was associated with downregulation of *ex vivo* synthesis of IL-17 and IFN-*γ* [101].

IL-35, also a member of the IL-12 family, is composed of EBI3 and p35. IL-35 is thought to be specifically produced by Tregs as a novel inhibitory cytokine and is required for maximal suppressive activity [102].

### 4.4. Transforming Growth Factor- (TGF-) *β*

TGF-*β* has variant roles due to the influence of the environments on its effects. DCs derived from tolerized mice, especially pDCs, produced increased levels of TGF-*β* and decreased levels of IL-6 after stimulation with nucleosomes, which favors the development of Tregs [79, 103]. TGF-*β* is unique among cytokines, because it can induce Foxp3 expression and aTregs differentiation in the absence of DCs [104].

### 4.5. Follicular Helper CD4 T (T_FH_) Cells

T_FH_ cells are important in the regulation of CD4 T cell lineage to Th1, Th2, Tregs, or Th17 cells [105]. T_FH_ cells expressed transcription factor Bcl6, which is a master regulator of T_FH_ differentiation. Bcl6 can antagonize transcription factors important for the differentiation of CD4 T cells to Th1, Th2, or Th17 cells. Bcl6 inhibits Th1 differentiation by binding to the *T-bet *gene, Th2 differentiation by inhibiting GATA3 protein and Th17 differentiation by inhibiting ROR*γ*t activity and the human ROR*γ*t promoter. Antagonism of Tregs by Bcl6 has not been reported, but gut Tregs lose Foxp3 and differentiate into Bcl6+ T_FH_ cells under inflammatory conditions [105]. T_FH_ cells seem to play important roles in common autoimmune diseases such as RA and SLE [106, 107]. T_FH_ cell pathway and effector molecules are potent therapeutic target candidates in SAIRDs.

### 4.6. B Cells

Natural autoreactive B cells specific for unmodified parts of self-antigens are normally present. In SLE patients, this autoreactive B cell fraction is probably larger than in healthy individuals [108] and a large proportion of B cells express TLR9, which correlates with high titers of anti-DNA autoantibodies [109]. In murine B cells, TLR9-MyD88-dependent signaling is critical for the class switch to pathogenic IgG antibodies [110]. Thus, at least in B cells, TLR9 with the BCR signaling appear to activate B cells and promote autoimmunity.

### 4.7. Skin Dendritic Cells (Skin DCs)

Psoriasis is a common chronic inflammatory disease of the skin in which the local activation of autoimmune DCs and T cells induces an abnormal differentiation of epidermal keratinocytes [111, 112]. TNF-**α**  and inducible nitric oxide synthase (iNOS) producing dermal CD11c+ DCs, so called TIP-DCs, are thought to be the human equivalent of a similar DC subset necessary for the clearance of some pathogens in mice [113]. In addition, also pDCs are increased in psoriatic skin compared with normal skin [114]. Infiltration of pDCs into psoriatic skin and their activation to produce type I IFN represent key upstream events that initiate the activation of autoimmune T cells, leading to the formation of the typical skin lesions [115]. 

LL37, an endogenous antimicrobial peptide, which is overexpressed in psoriatic skin, seems to be the key mediator of pDCs activation in psoriasis. LL37 breaks innate self-tolerance by forming a complex with self-DNA that is delivered to and retained within early endocytic compartments of pDCs to trigger TLR9 to induce type I IFN production, which then activates IFN signature [116]. LL 37 also binds self-RNA forming a complex which stimulates pDCs through TLR7 [117]. Self-RNA-LL37 complexes also interact with TLR8 on cDCs and promote their differentiation into mature DCs, which secrete TNF-**α**  and IL-6 [117]. LL37 is released during skin injury, breaks innate tolerance to self-DNA and self-RNA so that they can act as “danger signals” that potently activate innate antiviral-like immune responses through activation of endosomal TLRs in DCs [116, 117].

Recently, Vit. D3-activated epidermal Langerhans cell have been shown to induce the development of either TGF-*β*-dependent Foxp3+ Treg or IL-10-dependent IL-10+ Treg. This may be the mechanism via which Vit. D3 exerts its immunosuppressive function in inflammatory skin diseases [118]. In addition, Vit. D3 is thought to exert immunoregulation of DCs and T cells via the upregulation of CTLA-4.

## 5. Potential Treatment Options Based on the Modulation of Immune-Regulatory Processes (Table 1)

### 5.1. Glucocorticosteroids-Induced Immune Regulation

Glucocorticosteroids were the first immunosuppressants, which were used in the clinic. The inhibitory effect of glucocorticosteroids on the canonical NF-*κ*B pathway probably plays a key role in the generation of mature DCs and cytotoxic T cells [119].

Nonspecific immunosuppressive drugs such as glucocorticosteroids have numerous adverse effects and their use is sometimes limited by a lack of efficacy [120]. Specific interference in the production of cytokines, intracellular signaling pathway, autologous stem-cell transplantation, gene therapy, immune regulation-induced antigen therapy and DC vaccination are promising treatment targets for induction of SAIRDs at present.

### 5.2. Anticytokine Therapy

Therapies with biological agents to attain anti-inflammatory and immunosuppressive actions, using drugs such as anticytokines and cytokine receptors, have remarkably improved the results of the treatments for many SAIRDs patients [5, 12]. Successful identification of biological targets and their therapeutic translation (anti-TNF, anti-IL-6 receptor, anti-CD20, CTLA4-Ig, and anti-IL-1) will help many patients refractory to conventional intervention for RA, PsA, Crohn's disease, SLE, and so on [121]. Concentrations of proinflammatory cytokines, such as TNF-*α*, IL-1, IL-6, IL-12, IL-15, IL-18, type I IFN, IFN-*γ*, and B cell stimulating agents, are increased in SAIRDs. They play important roles in the inflammatory processes that lead to tissue and organ damage [122].

### 5.3. Tumor Necrosis Factor (TNF)

TNF-*α* stimulates migration of mature cDCs to the draining lymph nodes. TNF-*α* blockade can prevent this influx of antigen-presenting DCs to secondary lymphatic tissue, which is an important step for the activation of T cell responses [123]. The TNF blockers have been successfully used in the management of RA, PsA, and Crohn's disease; however, they can in some cases induce autoantibodies and lupus-like syndromes. Thus, their use in SLE is controversial [122, 124–126]. In SLE, the use of anti-TNF blockers has been associated with polyarthritis, cutaneous manifestations, disease activity, proteinuria, and nephritis but also severe infusion reactions [127, 128]. In SjS, anti-TNF blockers have not shown any clinical efficacy [129].

### 5.4. Interleukine- (IL-)1 and IL-18

Blockade of IL-1 and IL-18 has raised interest in human SLE. The results of two small, open-label trials of the recombinant human IL-1 receptor antagonist anakinra in SLE have been published, both of which reported beneficial effects [7, 130]. In RA, the effectiveness of IL-1 receptor antagonist therapy is clearly lower than that of the TNF blockers [131]. IL-18 acts as a chemoattractant stimulating the migration of pDCs to the glomeruli in the kidney [78] so its blockade might be a good therapeutic target. 

### 5.5. Interleukine (IL)-6

IL-6 is a pleiotropic cytokine that is overexpressed in patients with several SAIRDs. In contrast to other cytokines, IL-6 can bind to a soluble IL-6 receptor (sIL-6R) without being inhibited. On the contrary, this IL-6/sIL-6R complex can bind to IL-6R-negative cells via a nonligand binding but signal transducing pg130 component of the receptor complex. Therefore, antibodies against IL-6R inhibit such IL-6 actions. Accordingly, good clinical results were reported on the use of a humanized antibody against IL-6R, tocilizumab, in RA and juvenile idiopathic arthritis (JIA) [132]. An open-label study conducted using tocilizumab in SLE reported moderate effectiveness [133]. Some phase II trials of IL-6 blocking monoclonal antibodies are ongoing in SLE [134].

### 5.6. Interleukine (IL)-10

Disease activity in many SAIRDs has been considered to be driven by an imbalance between proinflammatory and anti-inflammatory cytokines. IL-10 is a powerful anti-inflammatory cytokine, which can be produced by both leukocytes and structural cells within tissues, being produced in particular by Tregs *in vivo* [20, 135]. However, high serum levels of IL-10 have been reported in patients with SLE and they correlated positively with the disease activity [136]. Therefore, somewhat paradoxically, in an open-label pilot study, a single injection of a mouse anti-IL-10 monoclonal antibody, given to a small group of active SLE patients, seemed to have beneficial clinical effects [137]. It was concluded that it was beneficial to boost the cell-mediated immune responses in SLE by inhibiting IL-10, because IL-10 impairs antigen presentation and Th1 lymphocyte activation.

### 5.7. Interleukine- (IL-) 17 and Others

IL-17 (or actually IL-17A) has been discovered to be a powerful proinflammatory cytokine and the recent detection of a Th17 T-helper cell subset that secretes it has focused attention on the role of IL-17 and Th17 cells in SAIRDs, in particular RA, PsA, multiple sclerosis and Crohn's disease, which are considered to represent Th17-related diseases. Several therapeutically interesting compounds have been reported, including anti-IL-17A agent (AIN 457 and LU2439821), anti-IL-17 receptor (AMG827) or anti-IL-17A/F receptor [92, 93, 138].

IL-12 and IL-23, which belongs to the IL-12 family, are important regulators of Th17 lymphocytes and dominant candidates for the treatment of SAIRDs with IL-17 involvement [139–141]. Anti-IL-12/23p40 agents (CNTO1275: Ustekinumab and ABT-874) are directed against both IL-12 and IL-23. An ustekinumab study is ongoing in phase III in PsA [142]. In addition, IL-21 and IL-22 are attractive therapeutic targets as cytokines related to Th17 cells [7, 122, 143]. IL-27 and IL-35 also belong to the IL-12 family and may be candidate tools to inhibit Th17 cells in the future [101, 102]. 

### 5.8. B Cell Related Cytokine

B-cell activating factor (BAFF, also known as B-lymphocyte stimulator (BLyS) and TNF ligand superfamily member 13B) promotes B-cell survival and autoantibody production. BAFF blocker (Belimumab) seems to be close to the public authority approval as they have been completed in large, double-blind, placebo-controlled studies of patients with active SLE in a phase III study [7, 144], completed in phase II study in RA [145]. Anti-CD22 monoclonal antibody (Epratuzumab) has finished a phase III trial in SLE [146].

Recent experimental and clinical evidence obtained in SSc-like mouse models and SSc patients suggests a role for B cells in the development of inflammation and fibrosis characteristic for this disease [147]. Antihuman CD20 antibody, that has been reported to be effective in the treatment of RA (Rituximab and Ofatumumab in phase III), had some beneficial effects also on skin fibrosis and lung involvement in SSc patients [148, 149]. In SjS patients, a phase II trial of Rituximab has been finished and Belimumab prepared [150, 151]. BAFF blocker as well as anti-CD20 blocker is one of potential drugs in the treatment of SAIRDs.

### 5.9. Intracellular Signaling Pathway

Among various signaling molecules activated by cytokine-receptor interaction, the small molecules targeting in particular Janus kinase- (JAK-) STAT pathway form attractive candidate drugs in the treatment of SAIRDs. Inhibitors of various JAK molecules (JAK-1/2, JAK-2, JAK-3) might be useful in the treatment of SAIRDs [5, 152, 153]. Some trials of JAK-3 inhibitor in patients with RA (CP690,550 in phase III), PsA and inflammatory bowel diseases have already been published [153–156]. JAK-3 is critical for signal transduction for IL-2, IL-4, IL-7, IL-9, IL-15, and IL-21, and a selective inhibition of JAK-3 has immunomodulatory effects, which affect T cells, B cells, macrophages, and NK cells, without significantly affecting other organ systems. JAK inhibitors may find clinical applications in SAIRDs in the near future. 

Spleen tyrosine kinase (SyK), which is another nonreceptor tyrosine kinase, could to be one of treatment targets in SAIRDs. A phase III trial of SyK inhibitor (R788) in RA (OSKIRA trial) is ongoing [157].

In addition to monoclonal antibodies and receptor fusion proteins, other alternative approaches to neutralize inflammatory cytokines are being developed in experimental models. These include vaccinations against cytokines, which have been found to be important in the disease mechanisms [158] and administration of small interfering RNAs (siRNAs) that target the messenger RNAs encoding key cytokines [3]. Another approach is to use small molecules to block certain cytokine pathways. For example, T-cell receptor (TCR) vaccinations and epitope-mimetic peptides (e.g., A9 in collagen-induced arthritis) are also in experimental use [159, 160].

### 5.10. Stem Cells and Immune Regulation

At first, most of the experience with autologous stem-cell transplantation in arthritis derived from studies with SSc, multiple sclerosis, and systemic JIA that were refractory to conventional therapies. Recently, also patients of RA and SLE have been treated with autologous hematopoietic stem cells, but their effect was limited in RA. Phase II trials of autologous stem-cell transplantation are ongoing in SLE and SSc [161]. In this procedure, autologous hematopoietic stem cells are collected and stored but transferred back to the donor after the remaining bone marrow and blood cells have been destroyed. The autotransplant then reconstitutes the hematopoietic and immune system and is hoped to restore the self-tolerance. Indeed, a small study reported that Tregs were restored by transplantation of autologous hematopoietic stem cells [162]. Autologous stem-cell transplantation therapy has potential in the treatment of also other SAIRDs in the future.

### 5.11. Gene Therapy

Some small clinical trials of the gene therapy have been done in RA *in vivo* and *ex vivo* [163]. Local viral gene therapies by tgACC 94, which is a recombinant adeno-associated virus serotype 2 vector genetically engineered to contain the cDNA for a human TNFR-immunoglobulin (IgG1) Fc fusion gene, are going on phase I in RA and phase II in PsA [164]. However, several reasons such as the limitation of efficacy and logistic and financial issues have deterred to step up the next trial phases. Technically insertions, alterations, or removals of genes can be done, but gene therapy is difficult to apply in polygenic diseases. Generation of an appropriate gene construct is already challenging but gene transfer and host immune responses against the gene therapy vectors form additional barriers to clinical applications. Due to the haphazard and random location of the gene in the host genome, insertional mutagenesis, and cancer development pose a potential threat. Further studies are needed for the gene therapy with new techniques such as siRNA [163, 165].

### 5.12. Immune Regulation Induced by Antigens

Secondary antigens (T-cell epitopes) that are involved in the amplification and maintenance of immune-inflammation, but independent of the initial and evasive “factor X” triggering the SAIRDs, might have therapeutic potential. Heat shock proteins (HSPs) are often targeted by proinflammatory T-cell responses in arthritis and induction of mucosal tolerance induced against their proinflammatory, immunodominant epitopes could provide a means to alleviate autoimmune inflammation in RA [166]. The dnaJP protein, which encodes a 15mer peptide (dnaJP1) derived from the bacterial HSP, was administered to induce immune tolerance in RA patients in a small trial study [167].

### 5.13. DC Therapy

Maintenance of the immature, immunoincompetent state of DCs, especially after *in vivo* delivery, remains a challenging but also promising task. For progress in this field, the methods for generation and delivery of tolerogenic or vaccinated DCs have first to be standardized.

Recently, chronic stimulation of pDCs with self-nucleosomes through TLR7 and TLR9 to produce type I IFN was reported to reduce therapeutic effects of glucocorticosteroids in SLE [90, 168]. *Vice versa*, inhibition of TLR7 improved disease manifestations in a lupus mouse model but not TLR9 [90, 91, 95]. 

The endogenous proapoptotic agents and the death receptors involved in the maturation of DCs and activation of specific T cell subsets, such as Th 17 cells, may become novel targets for the individualized, disease process-specific treatments of SAIRDs. Human monocyte-derived tolerogenic DCs, generated with dexamethasone and Vit. D3, maintained their tolerogenic function upon activation with lipopolysaccharide (LPS-tolerogenic DCs), while acquiring the ability to present human type II collagen (autoantigen) and migrate in response to CCR 7 ligand, CCL 19 [169, 170]. Vaccination with autologous tolerogenic DCs (RHEUMAVAX) is in preliminary phase I human trial [171].

## 6. Conclusion

The pathogenesis and mechanism of SAIRDs are not fully understood. However, various molecules, signal transduction, and immune effector pathways able to regulate immune regulation and tolerance are being increasingly revealed. For the generation of new drugs and therapies, it is important to know molecules and understand mechanisms responsible for the maintenance, failure, and restoration of tolerance in SAIRDs. Molecularly targeted and highly specific biologic agents have caused a paradigm shift of the treatment. However, in spite of top-of-the-line drugs applied according to current management strategies, some 20%–30% the patients do not respond adequately. Further, there are still several issues to be resolved, also with the currently used drugs, such as severe adverse events and the high cost although small chemical molecules, such as JAK inhibitors, might offer low cost options possible to administer easily per os. It is expected that the development leads to new drugs which are able to re-establish autoimmune regulation and tolerance in SAIRDs, drugs, which are more effective and tailored based on genetic polymorphism and at the same time safer, low cost, and easy to administer.

## Figures and Tables

**Table 1:** Potential treatment options for SAIRDs therapy.

	Immunogenicity	Immunoregultion and tolerance
Cytokine and receptor	TNF, TNFR	
	IL-1	IL-10?
	IL-2	IL-27
	IL-6, IL-6R	IL-35
	IL-12	
	IL-15	
	IL-17	
	IL-18	
	IL-21	
	IL-23	
	
	Type I IFN	TGF-*β*
	IFN-*γ*	
	
	BAFF	
	CD20	
	CD22	
	
	TCR (vaccination)	CTLA4-Ig
		
Intracellular signaling pathway	JAK-1	
	JAK-2	
	JAK-3	
	SyK	
Stem cells and immune regulation		Autologous stem cell transplantation
	
Gene therapy		TNFR: Fc
Immune regulation-induced antigen		HSPs?
DC therapy		Tolerogenic DC
		(DC vaccination)

TNF: tumor necrosis factor, TNFR: tumor necrosis factor receptor, IL: interleukine, R: receptor, IFN: interferon, TGF: transforming growth factor, BAFF: B-cell activating factor, CTLA: cytotoxic T-lymphocyte antigen, TCR: T cell receptor, JAK: Janus kinase, SyK: spleen tyrosine kinase, TNFR: Fc: soluble form of the tumor necrosis factor receptor, HSPs: heat shock proteins, and DC: dendritic cell.

## References

[1] Banchereau J, Steinman RM (1998). Dendritic cells and the control of immunity. *Nature*.

[2] Saurer L, Mueller C (2009). T cell-mediated immunoregulation in the gastrointestinal tract. *Allergy*.

[3] Hu N, Long H, Zhao M, Yin H, Lu Q (2009). Aberrant expression pattern of histone acetylation modifiers and mitigation of lupus by SIRT1-siRNA in MRL/*lpr* mice. *Scandinavian Journal of Rheumatology*.

[4] Hu J, Wan Y (2011). Tolerogenic dendritic cells and their potential applications. *Immunology*.

[5] Albani S, Koffeman EC, Prakken B (2011). Induction of immune tolerance in the treatment of rheumatoid arthritis. *Nature Reviews Rheumatology*.

[6] Firestein GS (2003). Evolving concepts of rheumatoid arthritis. *Nature*.

[7] Rönnblom L, Elkon KB (2010). Cytokines as therapeutic targets in SLE. *Nature Reviews Rheumatology*.

[8] Benson RA, Patakas A, Conigliaro P (2010). Identifying the cells breaching self-tolerance in autoimmunity. *Journal of Immunology*.

[9] Steinman RM, Banchereau J (2007). Taking dendritic cells into medicine. *Nature*.

[10] Jaen O, Rullé S, Bessis N, Zago A, Boissier MC, Falgarone G (2009). Dendritic cells modulated by innate immunity improve collagen-induced arthritis and induce regulatory T cells in vivo. *Immunology*.

[11] Jongbloed SL, Benson RA, Nickdel MB, Garside P, McInnes IB, Brewer JM (2009). Plasmacytoid dendritic cells regulate breach of self-tolerance in autoimmune arthritis. *Journal of Immunology*.

[12] Lebre MC, Tak PP (2009). Dendritic cells in rheumatoid arthritis: which subset should be used as a tool to induce tolerance?. *Human Immunology*.

[13] Takakubo Y, Takagi M, Maeda K (2008). Distribution of myeloid dendritic cells and plasmacytoid dendritic cells in the synovial tissues of rheumatoid arthritis. *Journal of Rheumatology*.

[14] Ginhoux F, Tacke F, Angeli V (2006). Langerhans cells arise from monocytes in vivo. *Nature Immunology*.

[15] Alvarez D, Vollmann EH, von Andrian UH (2008). Mechanisms and Consequences of Dendritic Cell Migration. *Immunity*.

[16] Seitz HM, Matsushima GK (2010). Dendritic cells in systemic lupus erythematosus. *International Reviews of Immunology*.

[17] Steinman RM, Idoyaga J (2010). Features of the dendritic cell lineage. *Immunological Reviews*.

[18] Kretschmer K, Apostolou I, Hawiger D, Khazaie K, Nussenzweig MC, von Boehmer H (2005). Inducing and expanding regulatory T cell populations by foreign antigen. *Nature Immunology*.

[19] Tsuji NM, Kosaka A (2008). Oral tolerance: intestinal homeostasis and antigen-specific regulatory T cells. *Trends in Immunology*.

[20] Maldonado RA, von Andrian UH (2010). How tolerogenic dendritic cells induce regulatory T cells. *Advances in Immunology*.

[21] Barton GM, Kagan JC, Medzhitov R (2006). Intracellular localization of Toll-like receptor 9 prevents recognition of self DNA but facilitates access to viral DNA. *Nature Immunology*.

[22] Moseman EA, Liang X, Dawson AJ (2004). Human plasmacytoid dendritic cells activated by CpG oligodeoxynucleotides induce the generation of CD4^+^CD25^+^ regulatory T cells. *Journal of Immunology*.

[23] Jin O, Kavikondala S, Mok MY (2010). Abnormalities in circulating plasmacytoid dendritic cells in patients with systemic lupus erythematosus. *Arthritis Research & Therapy*.

[24] Puccetti P, Grohmann U (2007). IDO and regulatory T cells: a role for reverse signalling and non-canonical NF-*κ*B activation. *Nature Reviews Immunology*.

[25] Mellor AL, Munn DH (2004). IDO expression by dendritic cells: tolerance and tryptophan catabolism. *Nature Reviews Immunology*.

[26] Curti A, Trabanelli S, Salvestrini V, Baccarani M, Lemoli RM (2009). The role of indoleamine 2,3-dioxygenase in the induction of immune tolerance: focus on hematology. *Blood*.

[27] Sakaguchi S, Miyara M, Costantino CM, Hafler DA (2010). FOXP3^+^ regulatory T cells in the human immune system. *Nature Reviews Immunology*.

[28] Tang Q, Bluestone JA (2008). The Foxp3^+^ regulatory T cell: a jack of all trades, master of regulation. *Nature Immunology*.

[29] Steinman RM, Hawiger D, Nussenzweig MC (2003). Tolerogenic dendritic cells. *Annual Review of Immunology*.

[30] Roncarolo M, Battaglia M (2007). Regulatory T-cell immunotherapy for tolerance to self antigens and alloantigens in humans. *Nature Reviews Immunology*.

[31] Cobbold SP, Adams E, Farquhar CA (2009). Infectious tolerance via the consumption of essential amino acids and mTOR signaling. *Proceedings of the National Academy of Sciences of the United States of America*.

[32] Menard L, Samuels J, Ng YS, Meffre E (2011). Inflammation-independent defective early B cell tolerance checkpoints in rheumatoid arthritis. *Arthritis and Rheumatism*.

[33] Anolik JH, Ravikumar R, Barnard J (2008). Cutting edge: anti-tumor necrosis factor therapy in rheumatoid arthritis inhibits memory B lymphocytes via effects on lymphoid germinal centers and follicular dendritic cell networks. *Journal of Immunology*.

[34] Pistoia V (1997). Production of cytokines by human B cells in health and disease. *Immunology Today*.

[35] Johansson-Lindbom B, Borrebaeck CA (2002). Germinal center B cells constitute a predominant physiological source of IL-4: implication for Th2 development in vivo. *Journal of Immunology*.

[36] Mauri C, Gray D, Mushtaq N, Londei M (2003). Prevention of arthritis by interleukin 10-producing B cells. *Journal of Experimental Medicine*.

[37] Lenert P, Brummel R, Field EH, Ashman RF (2005). TLR-9 activation of marginal zone B cells in lupus mice regulates immunity through increased IL-10 production. *Journal of Clinical Immunology*.

[38] Mizoguchi A, Mizoguchi E, Takedatsu H, Blumberg RS, Bhan AK (2002). Chronic intestinal inflammatory condition generates IL-10-producing regulatory B cell subset characterized by CD1d upregulation. *Immunity*.

[39] Mann MK, Maresz K, Shriver LP, Tan Y, Dittel BN (2007). B cell regulation of CD4^+^CD25^+^ T regulatory cells and IL-10 via B7 is essential for recovery from experimental autoimmune encephalomyelitis. *Journal of Immunology*.

[40] Lemoine S, Morva A, Youinou P, Jamin C (2009). Regulatory B cells in autoimmune diseases: how do they work. *Annals of the New York Academy of Sciences*.

[41] Mizoguchi A, Bhan AK (2006). A case for regulatory B cells. *Journal of Immunology*.

[42] Hughes LB, Reynolds RJ, Brown EE (2010). Most common single-nucleotide polymorphisms associated with rheumatoid arthritis in persons of European ancestry confer risk of rheumatoid arthritis in African Americans. *Arthritis and Rheumatism*.

[43] Stahl EA, Raychaudhuri S, Remmers EF (2010). Genome-wide association study meta-analysis identifies seven new rheumatoid arthritis risk loci. *Nature Genetics*.

[44] Nordmark G, Kristjansdottir G, Theander E (2011). Association of EBF1, FAM167A(C8orf13)-BLK and TNFSF4 gene variants with primary Sjögren's syndrome. *Genes and Immunity*.

[45] Lee YH, Bae SC, Choi SJ, Ji JD, Song GG (2011). Associations between vitamin D receptor polymorphisms and susceptibility to rheumatoid arthritis and systemic lupus erythematosus: a meta-analysis. *Molecular Biology Reports*.

[46] Raychaudhuri S, Remmers EF, Lee AT (2008). Common variants at CD40 and other loci confer risk of rheumatoid arthritis. *Nature Genetics*.

[47] MacGregor AJ, Snieder H, Rigby AS (2000). Characterizing the quantitative genetic contribution to rheumatoid arthritis using data from twins. *Arthritis and Rheumatism*.

[48] Lundströom E, Källberg H, Alfredsson L, Klareskog L, Padyukov L (2009). Gene-environment interaction between the DRB1 shared epitope and smoking in the risk of anti-citrullinated protein antibody-positive rheumatoid arthritis: all alleles are important. *Arthritis and Rheumatism*.

[49] van der Woude D, Houwing-Duistermaat JJ, Toes RE (2009). Quantitative heritability of anti-citrullinated protein antibody-positive and anti-citrullinated protein antibody-negative rheumatoid arthritis. *Arthritis and Rheumatism*.

[50] de Vries RR, van der Woude D, Houwing JJ, Toes RE (2011). Genetics of ACPA-positive rheumatoid arthritis: the beginning of the end?. *Annals of the Rheumatic Diseases*.

[51] Deighton CM, Walker DJ, Griffiths ID, Roberts DF (1989). The contribution of HLA to rheumatoid arthritis. *Clinical Genetics*.

[52] Niewold TB, Kelly JA, Flesch MH, Espinoza LR, Harley JB, Crow MK (2008). Association of the IRF5 risk haplotype with high serum interferon-*α* activity in systemic lupus erythematosus patients. *Arthritis and Rheumatism*.

[53] Kariuki SN, Kirou KA, MacDermott EJ, Barillas-Arias L, Crow MK, Niewold TB (2009). Cutting edge: autoimmune disease risk variant of *STAT4* confers increased sensitivity to IFN-*α* in lupus patients in vivo. *Journal of Immunology*.

[54] Adrianto I, Wen F, Templeton A (2011). Association of a functional variant downstream of TNFAIP3 with systemic lupus erythematosus. *Nature Genetics*.

[55] Pullmann R, Bonilla E, Phillips PE, Middleton FA, Perl A (2008). Haplotypes of the HRES-1 endogenous retrovirus are associated with development and disease manifestations of systemic lupus erythematosus. *Arthritis and Rheumatism*.

[56] Rahman P (2011). Current challenges in the genetics of Psoriatic arthritis: a report from the GRAPPA 2009 annual meeting. *Journal of Rheumatology*.

[57] Rahman P (2011). Update on genetics of psoriasis and prosiatic arthritis. *Annals of the Rheumatic Disease*.

[58] Radstake TR, Gorlova O, Rueda B (2010). Genome-wide association study of systemic sclerosis identifies CD247 as a new susceptibility locus. *Nature Genetics*.

[59] Silman SJ, Newman J, MacGregor AJ (1996). Cigarette smoking increases the risk of rheumatoid arthritis: results from a nationwide study of disease-discordant twins. *Arthritis and Rheumatism*.

[60] Lundberg K, Wegner N, Yucel-Lindberg T, Venables PJ (2010). Periodontitis in RA-the citrullinated enolase connection. *Nature Reviews Rheumatology*.

[61] Claas FH, Gijbels Y, van der Velden-de Munck J, van Rood JJ (1988). Induction of B cell unresponsiveness to noninherited maternal HLA antigens during fetal life. *Science*.

[62] Feitsma AL, Worthington J, van der Helm-van Mil AH (2007). Protective effect of noninherited maternal HLA-DR antigens on rheumatoid arthritis development. *Proceedings of the National Academy of Sciences of the United States of America*.

[63] Suzuki T, Katsunori I, Koichiro Y (2011). Genome wide association study identified ABCA1/SLC44A1 as risk loci for the joint destruction in Japanese RA patients. *Annals of the Rheumatic Disease*.

[64] Coenen MJ, Umićević M, Scheffer H (2011). Replication of loci from genome-wide association studies of anti-tumor necrosis factor treatment outcome in patients with rheumatoid arthritis. Results from the DREAM registry. *Annals of the Rheumatic Disease*.

[65] Knevel R, Wilson A, Daha N (2011). Polymorphisms in GZMB associate with rate of joint destruction in rheumatoid arthritis. *Annals of the Rheumatic Disease*.

[66] Cui J, Saevarsdottir S, Thomson B (2010). Rheumatoid arthritis risk allele PTPRC is also associated with response to anti-tumor necrosis factor *α* therapy. *Arthritis and Rheumatism*.

[67] Surolia I, Pirnie SP, Chellappa V (2010). Functionally defective germline variants of sialic acid acetylesterase in autoimmunity. *Nature*.

[68] Duroux-Richard I, Jorgensen C, Apparailly F (2011). miRNAs and rheumatoid arthritis—promising novel biomarkers. *Swiss Medical Weekly*.

[69] Stagakis E, Bertsias G, Verginis P (2011). Identification of novel microRNA signatures linked to human lupus disease activity and pathogenesis: miR-21 regulates aberrant T cell responses through regulation of PDCD4 expression. *Annals of the Rheumatic Diseases*.

[70] Hahn BH (1998). Antibodies to DNA. *New England Journal of Medicine*.

[71] Barrat FJ, Coffman RL (2008). Development of TLR inhibitors for the treatment of autoimmune diseases. *Immunological Reviews*.

[72] Lövgren T, Eloranta ML, Båve U, Alm GV, Rönnblom L (2004). Induction of interferon-*α* production in plasmacytoid dendritic cells by immune complexes containing nucleic acid released by necrotic or late apoptotic cells and lupus IgG. *Arthritis and Rheumatism*.

[73] Preble OT, Black RJ, Friedman RM, Klippel JH, Vilcek J (1982). Systemic lupus erythematosus: presence in human serum of an unusual acid-labile leukocyte interferon. *Science*.

[74] Farkas L, Beiske K, Lund-Johansen F, Brandtzaeg P, Jahnsen FL (2001). Plasmacytoid dendritic cells (natural interferon-*α*/*β*-producing cells) accumulate in cutaneous lupus erythematosus lesions. *American Journal of Pathology*.

[75] Rönnblom L, Alm GV (2001). A pivotal role for the natural interferon *α*-producing cells (plasmacytoid dendritic cells) in the pathogenesis of lupus. *Journal of Experimental Medicine*.

[76] Rönnblom L, Eloranta ML, Alm GV (2003). Role of natural interferon-*α* producing cells (plasmacytoid dendritic cells) in autoimmunity. *Autoimmunity*.

[77] Means TK, Latz E, Hayashi F, Murali MR, Golenbock DT, Luster AD (2005). Human lupus autoantibody-DNA complexes activate DCs through cooperation of CD32 and TLR9. *Journal of Clinical Investigation*.

[78] Tucci M, Quatraro C, Lombardi L, Pellegrino C, Dammacco F, Silvestris F (2008). Glomerular accumulation of plasmacytoid dendritic cells in active lupus nephritis: role of interleukin-18. *Arthritis and Rheumatism*.

[79] Fransen JH, Hilbrands LB, Ruben J (2009). Mouse dendritic cells matured by ingestion of apoptotic blebs induce T cells to produce interleukin-17. *Arthritis and Rheumatism*.

[80] Theofilopoulos AN, Baccala R, Beutler B, Kono DH (2005). Type I interferons (*α*/*β*) in immunity and autoimmunity. *Annual Review of Immunology*.

[81] Kyogoku C, Tsuchiya N (2007). A compass that points to lupus: genetic studies on type I interferon pathway. *Genes & Immunity*.

[82] Hagberg N, Berggren O, Leonard D (2011). IFN-*α* production by plasmacytoid dendritic cells stimulated with RNA-containing immune complexes is promoted by NK cells via MIP-1*β* and LFA-1. *Journal of Immunology*.

[83] Amoura Z, Koutouzov S, Piette JC (2000). The role of nucleosomes in lupus. *Current Opinion in Rheumatology*.

[84] Urbonaviciute V, Fürnrohr BG, Meister S (2008). Induction of inflammatory and immune responses by HMGB1-nucleosome complexes: implications for the pathogenesis of SLE. *Journal of Experimental Medicine*.

[85] Willingham SB, Allen IC, Bergstralh DT (2009). NLRP3 (NALP3, cryopyrin) facilitates in vivo caspase-1 activation, necrosis, and HMGB1 release via inflammasome-dependent and -independent pathways. *Journal of Immunology*.

[86] Liu G, Wang J, Park YJ (2008). High mobility group protein-1 inhibits phagocytosis of apoptotic neutrophils through binding to phosphatidylserine. *Journal of Immunology*.

[87] Sturfelt G, Truedsson L (2005). Complement and its breakdown products in SLE. *Rheumatology*.

[88] Chitrabamrung S, Rubin RL, Tan EM (1981). Serum deoxyribonuclease I and clinical activity in systemic lupus erythematosus. *Rheumatology International*.

[89] Lech M, Kulkarni OP, Pfeiffer S (2008). Tir8/Sigirr prevents murine lupus by suppressing the immunostimulatory effects of lupus autoantigens. *Journal of Experimental Medicine*.

[90] Christensen SR, Shupe J, Nickerson K, Kashgarian M, Flavell RA, Shlomchik MJ (2006). Toll-like receptor 7 and TLR9 dictate autoantibody specificity and have opposing inflammatory and regulatory roles in a murine model of lupus. *Immunity*.

[91] Wu X, Peng SL (2006). Toll-like receptor 9 signaling protects against murine lupus. *Arthritis and Rheumatism*.

[92] Korn T, Bettelli E, Oukka M, Kuchroo VK (2009). IL-17 and Th17 cells. *Annual Review of Immunology*.

[93] Wong CK, Lit LC, Tam LS, Li EK, Wong PT, Lam CW (2008). Hyperproduction of IL-23 and IL-17 in patients with systemic lupus erythematosus: implications for Th17-mediated inflammation in auto-immunity. *Clinical Immunology*.

[94] Pasare C, Medzhitov R (2003). Toll pathway-dependent blockade of CD4+CD25+ T cell-mediated suppression by dendritic cells. *Science*.

[95] Fransen JH, van der Vlag J, Ruben J, Adema GJ, Berden JH, Hilbrands LB (2010). The role of dendritic cells in the pathogenesis of systemic lupus erythematosus. *Arthritis Research & Therapy*.

[96] Liu MF, Wang CR, Fung LL, Wu CR (2004). Decreased CD4^+^CD25^+^ T cells in peripheral blood of patients with systemic lupus erythematosus. *Scandinavian Journal of Immunology*.

[97] Valencia X, Stephens G, Goldbach-Mansky R, Wilson M, Shevach EM, Lipsky PE (2006). TNF downmodulates the function of human CD4^+^CD25^hi^ T-regulatory cells. *Blood*.

[98] Xiao S, Jin H, Korn T (2008). Retinoic acid increases Foxp3^+^ regulatory T cells and inhibits development of Th17 cells by enhancing TGF-*β*-driven Smad3 signaling and inhibiting IL-6 and IL-23 receptor expression. *Journal of Immunology*.

[99] Kwok SK, Cho ML, Moon SJ (2011). Retinoic acid attenuates inflammatory arthritis by reciprocal regulation of Th17 and regulatory T cells and by inhibiting osteoclastogenesis in an autoimmune arthritis model. *Annals of the Rheumatic Disease*.

[100] Goldberg R, Wildbaum G, Zohar Y, Maor G, Karin N (2004). Suppression of ongoing adjuvant-induced arthritis by neutralizing the function of the p28 subunit of IL-27. *Journal of Immunology*.

[101] Niedbala W, Cai B, Wei X (2008). Interleukin 27 attenuates collagen-induced arthritis. *Annals of the Rheumatic Diseases*.

[102] Collison LW, Workman CJ, Kuo TT (2007). The inhibitory cytokine IL-35 contributes to regulatory T-cell function. *Nature*.

[103] Kang HK, Liu M, Datta SK (2007). Low-dose peptide tolerance therapy of lupus generates plasmacytoid dendritic cells that cause expansion of autoantigen-specific regulatory T cells and contraction of inflammatory Th17 cells. *Journal of Immunology*.

[104] Chen W, Jin W, Hardegen N (2003). Conversion of peripheral CD4^+^CD25^−^ Naive T Cells to CD4^+^CD25^+^ Regulatory T Cells by TGF-*β* induction of transcription factor foxp3. *Journal of Experimental Medicine*.

[105] Crotty S (2011). Follicular Helper CD4 T cells (T_FH_). *Annual Review of Immunology*.

[106] Linterman MA, Rigby RJ, Wong RK (2009). Follicular helper T cells are required for systemic autoimmunity. *Journal of Experimental Medicine*.

[107] Simpson N, Gatenby PA, Wilson A (2010). Expansion of circulating T cells resembling follicular helper T cells is a fixed phenotype that identifies a subset of severe systemic lupus erythematosus. *Arthritis and Rheumatism*.

[108] Yurasov S, Wardemann H, Hammersen J (2005). Defective B cell tolerance checkpoints in systemic lupus erythematosus. *Journal of Experimental Medicine*.

[109] Papadimitraki ED, Choulaki C, Koutala E (2006). Expansion of toll-like receptor 9-expressing B cells in active systemic lupus erythematosus: implications for the induction and maintenance of the autoimmune process. *Arthritis and Rheumatism*.

[110] Ehlers M, Fukuyama H, McGaha TL, Aderem A, Ravetch JV (2006). TLR9/MyD88 signaling is required for class switching to pathogenic IgG2a and 2b autoantibodies in SLE. *Journal of Experimental Medicine*.

[111] Nickoloff BJ, Nestle FO (2004). Recent insights into the immunopathogenesis of psoriasis provide new therapeutic opportunities. *Journal of Clinical Investigation*.

[112] Lowes MA, Bowcock AM, Krueger JG (2007). Pathogenesis and therapy of psoriasis. *Nature*.

[113] Lande R, Gregorio J, Facchinetti V (2007). Plasmacytoid dendritic cells sense self-DNA coupled with antimicrobial peptide. *Nature*.

[114] Lowes MA, Chamian F, Abello MV (2005). Increase in TNF-*α* and inducible nitric oxide synthase-expressing dendritic cells in psoriasis and reduction with efalizumab (anti-CD11a). *Proceedings of the National Academy of Sciences of the United States of America*.

[115] Nestle FO, Conrad C, Tun-Kyi A (2005). Plasmacytoid predendritic cells initiate psoriasis through interferon-*α* production. *Journal of Experimental Medicine*.

[116] Nestle FO, di Meglio P, Qin JZ, Nickoloff BJ (2009). Skin immune sentinels in health and disease. *Nature Reviews Immunology*.

[117] Ganguly D, Chamilos G, Lande R (2009). Self-RNA-antimicrobial peptide complexes activate human dendritic cells through TLR7 and TLR8. *Journal of Experimental Medicine*.

[118] van der Aar AMG, Sibiryak DS, Bakdash G (2011). Vitamin D3 targets epidermal and dermal dendritic cells for induction of distinct regulatory T cells. *Journal of Allergy and Clinical Immunology*.

[119] Ito K, Chung KF, Adcock IM (2006). Update on glucocorticoid action and resistance. *Journal of Allergy and Clinical Immunology*.

[120] Yoo DH (2010). Anticytokine therapy in systemic lupus erythematosus. *Lupus*.

[121] Feldmann M (2002). Development of anti-TNF therapy for rheumatoid arthritis. *Nature Reviews Immunology*.

[122] La Cava A (2010). Anticytokine therapies in systemic lupus erythematosus. *Immunotherapy*.

[123] Itano AA, McSorley SJ, Reinhardt RL (2003). Distinct dendritic cell populations sequentially present antigen to CD4 T cells and stimulate different aspects of cell-mediated immunity. *Immunity*.

[124] Charles P, Smeenk R, de Jong J, Feldmann M, Maini R (2000). Assessment of antibodies to double-stranded DNA induced in rheumatoid arthritis patients following treatment with infliximab, a monoclonal antibody to tumor necrosis factor *α*: findings in open-label and randomized placebo-controlled trials. *Arthritis and Rheumatism*.

[125] de Rycke L, Kruithof E, van Damme N (2003). Antinuclear antibodies following infliximab treatment in patients with rheumatoid arthritis or spondylarthropathy. *Arthritis and Rheumatism*.

[126] Shakoor N, Michalska M, Harris C, Block J (2002). Drug-induced systemic lupus erythematosus associated with etanercept therapy. *Lancet*.

[127] Aringer M, Steiner G, Graninger W, Höfler E, Steiner CW, Smolen JS (2007). Effects of short-term infliximab therapy on autoantibodies in systemic lupus erythematosus. *Arthritis and Rheumatism*.

[128] Hayat S, Uppal S (2007). Therapeutic efficacy and safety profile of infliximab in active systemic lupus erythematosus. *Modern Rheumatology*.

[129] Mariette X, Ravaud P, Steinfeld S (2004). Inefficacy of infliximab in primary Sjögren's syndrome: results of the randomized, controlled Trial of remicade in primary Sjögren's syndrome (TRIPSS). *Arthritis and Rheumatism*.

[130] Ostendorf B, Iking-Konert C, Kurz K, Jung G, Sander O, Schneider M (2005). Preliminary results of safety and efficacy of the interleukin 1 receptor antagonist anakinra in patients with severe lupus arthritis. *Annals of the Rheumatic Diseases*.

[131] Buch MH, Bingham SJ, Seto Y (2004). Lack of response to anakinra in rheumatoid arthritis following failure of tumor necrosis factor *α* blockade. *Arthritis and Rheumatism*.

[132] Smolen JS, Beaulieu A, Rubbert-Roth A (2008). Effect of interleukin-6 receptor inhibition with tocilizumab in patients with rheumatoid arthritis (OPTION study): a double-blind, placebo-controlled, randomised trial. *Lancet*.

[133] Illei GG, Shirota Y, Yarboro CH (2010). Tocilizumab in systemic lupus erythematosus: data on safety, preliminary efficacy, and impact on circulating plasma cells from an open-label phase I dosage-escalation study. *Arthritis and Rheumatism*.

[134] http://www.clinicaltrials.gov/ct2/results?term=IL-6+lupus.

[135] Wakkach A, Augier S, Breittmayer JP, Blin-Wakkach C, Carle GF (2008). Characterization of IL-10-secreting T cells derived from regulatory CD4^+^CD25^+^ cells by the TIRC7 surface marker. *Journal of Immunology*.

[136] Park YB, Lee SK, Kim DS, Lee J, Lee CH, Song CH (1998). Elevated interleukin-10 levels correlated with disease activity in systemic lupus erythematosus. *Clinical and Experimental Rheumatology*.

[137] Llorente L, Richaud-Patin Y, García-Padilla C (2000). Clinical and biologic effects of anti-interleukin-10 monoclonal antibody administration in systemic Lupus erythematosus. *Arthritis and Rheumatism*.

[138] Genovese MC, van den Bosch F, Roberson SA (2010). LY2439821, a humanized anti-interleukin-17 monoclonal antibody, in the treatment of patients with rheumatoid arthritis: a phase I randomized, double-blind, placebo-controlled, proof-of-concept study. *Arthritis and Rheumatism*.

[139] Young DA, Hegen M, Ma HL (2007). Blockade of the interleukin-21/interleukin-21 receptor pathway ameliorates disease in animal models of rheumatoid arthritis. *Arthritis and Rheumatism*.

[140] Melis L, Vandooren B, Kruithof E (2010). Systemic levels of IL-23 are strongly associated with disease activity in rheumatoid arthritis but not spondyloarthritis. *Annals of the Rheumatic Diseases*.

[141] Kurzeja M, Rudnicka L, Olszewska M (2011). New interleukin-23 pathway inhibitors in dermatology: ustekinumab, briakinumab, and secukinumab. *American Journal of Clinical Dermatology*.

[142] http://www.clinicaltrials.gov/ct2/show/NCT01090427?term=Ustekinumab&rank=7.

[143] Kimball AB, Gordon KB, Langley RG, Menter A, Perdok RJ, Valdes J (2011). ABT-874 Study Investigators. Efficacy and safety of ABT-874, a monoclonal anti-interleukin 12/23 antibody, for the treatment of chronic plaque psoriasis: 36-week observation/retreatment and 60-week open-label extension phases of a randomized phase II trial. *Journal of American Academy Dermatology*.

[144] http://www.clinicaltrials.gov/ct2/show/NCT00410384?term=Belimumab+lupus&rank=1.

[145] http://www.clinicaltrials.gov/ct2/show/NCT00071812?term=belimumab+arthritis&rank=2.

[146] http://www.clinicaltrials.gov/ct2/show/NCT01262365?term=Epratuzumab+lupus&rank=1.

[147] Bosello S, de Luca G, Tolusso B (2011). B cells in systemic sclerosis: a possible target for therapy. *Autoimmunity Reviews*.

[148] Smith V, van Praet JT, Vandooren B (2010). Rituximab in diffuse cutaneous systemic sclerosis: an open-label clinical and histopathological study. *Annals of the Rheumatic Diseases*.

[149] Taylor PC, Quattrocchi E, Mallett S (2011). Ofatumumab, a fully human anti-CD20 mAb, in the treatment of biologic-naïve rheumatoid arthritis patients: a rndomised, double-blind, placebo-controlled clinical trial. *Annals of the Rheumatic Disease*.

[150] http://www.clinicaltrials.gov/ct2/show/NCT00426543?term=BAFF+rheumatic&rank=3.

[151] http://www.clinicaltrials.gov/ct2/show/NCT01160666?term=belimumab+arthritis&rank=9.

[152] Cohen S, Fleischmann R (2010). Kinase inhibitors: a new approach to rheumatoid arthritis treatment. *Current Opinion in Rheumatology*.

[153] Kremer JM, Bloom BJ, Breedveld FC (2009). The safety and efficacy of a JAK inhibitor in patients with active rheumatoid arthritis: results of a double-blind, placebo-controlled phase IIa trial of three dosage levels of CP-690,550 versus placebo. *Arthritis and Rheumatism*.

[154] Boy MG, Wang C, Wilkinson BE (2009). Double-blind, placebo-controlled, dose-escalation study to evaluate the pharmacologic effect of CP-690,550 in patients with psoriasis. *Journal of Investigative Dermatology*.

[155] Monteleone G, Pallone F, Macdonald TT, Chimenti S, Costanzo A (2011). Psoriasis: from pathogenesis to novel therapeutic approaches. *Clinical Science*.

[156] http://www.clinicaltrials.gov/ct2/show/NCT00814307?term=CP690%E3%80%80RA%E3%80%80phase+III&rank=4.

[157] http://www.clinicaltrials.gov/ct2/show/NCT01197534?term=OSKIRA+rheumatic&rank=1.

[158] Zagury D, Buanec HL, Mathian A (2009). IFN*α* kinoid vaccine-induced neutralizing antibodies prevent clinical manifestations in a lupus flare murine model. *Proceedings of the National Academy of Sciences of the United States of America*.

[159] St Clair EW (2009). Novel targeted therapies for autoimmunity. *Current Opinion in Immunology*.

[160] Tang B, Zhou J, Park JE (2009). T cell receptor signaling induced by an analog peptide of type II collagen requires activation of Syk. *Clinical Immunology*.

[161] Farge D, Labopin M, Tyndall A (2010). Autologous hematopoietic stem cell transplantation for autoimmune diseases: an observational study on 12 years' experience from the European Group for Blood and Marrow Transplantation Working Party on Autoimmune Diseases. *Haematologica*.

[162] de Kleer I, Vastert B, Klein M (2006). Autologous stem cell transplantation for autoimmunity induces immunologic self-tolerance by reprogramming autoreactive T cells and restoring the CD4^+^CD25^+^ immune regulatory network. *Blood*.

[163] Jorgensen C, Apparailly F (2010). Prospects for gene therapy in inflammatory arthritis. *Best Practice and Research*.

[164] http://www.clinicaltrials.gov/ct2/show/NCT00902486?term=rheumatic+drug&rank=3.

[165] Colella P, Cotugno G, Auricchio A (2009). Ocular gene therapy: current progress and future prospects. *Trends in Molecular Medicine*.

[166] de Jong H, Jager W, Haverkamp MH (2009). Pan-DR-binding Hsp60 self epitopes induce an interleukin-10-mediated immune response in rheumatoid arthritis. *Arthritis and Rheumatism*.

[167] Koffeman EC, Genovese M, Amox D (2009). Epitope-specific immunotherapy of rheumatoid arthritis: clinical responsiveness occurs with immune deviation and relies on the expression of a cluster of molecules associated with T cell tolerance in a double-blind, placebo-controlled, pilot phase II trial. *Arthritis and Rheumatism*.

[168] Guiducci C, Gong M, Xu Z (2010). TLR recognition of self nucleic acids hampers glucocorticoid activity in lupus. *Nature*.

[169] Anderson AE, Swan DJ, Sayers BL (2009). LPS activation is required for migratory activity and antigen presentation by tolerogenic dendritic cells. *Journal of Leukocyte Biology*.

[170] Stoop JN, Harry RA, von Delwig A, Isaacs JD, Robinson JH, Hilkens CM (2010). Therapeutic effect of tolerogenic dendritic cells in established collagen-induced arthritis is associated with a reduction in Th17 responses. *Arthritis and Rheumatism*.

[171] Thomas R, Street S, Ramnoruth N (2011). Safety and preliminary evidence of efficacy in a phase I clinical trial of autologous tolerisng dendritic cells exposed to citrullinated peptides (RHEUMAVAX) in patients with rheumatoid arthritis. *Annals of the Rheumatic Disease*.

